# Oxidative Molecular Mechanisms Underlying Liver Diseases: From Systems Biology to the Personalized Medicine

**DOI:** 10.1155/2019/7864316

**Published:** 2019-06-02

**Authors:** Daniele Vergara, Andrea Casadei-Gardini, Anna M. Giudetti

**Affiliations:** ^1^Department of Biological and Environmental Sciences and Technologies, University of Salento, Lecce, Italy; ^2^Division of Medical Oncology, Department of Medical and Surgical Sciences for Children and Adults, University Hospital of Modena, Italy

The liver is the second largest organ in the body with a major contribution to the regulation of systemic metabolism. This places the liver at the centre of an extraordinary biomedical interest due to the increased prevalence worldwide of liver-associated diseases and due to the complex molecular background behind these conditions. Moreover, the liver plays a key role in the control of whole-body energy through the physiological regulation of different metabolisms including that of sugars, lipids, and amino acids. The alteration of liver metabolic homeostasis is critical for the development and progression of different diseases including nonalcoholic fatty liver disease (NAFLD), nonalcoholic steatohepatitis (NASH), and hepatocellular carcinoma (HCC). These circumstances generate a fertile ground for the definition of altered cellular and molecular functions at the basis of these conditions. To further increase our understanding of this complexity, this special issue provides an overview of these processes with potential advances in the development of diagnostic and clinical applications.

Fatty liver is considered a consequence of a higher flux of nonesterified fatty acids derived from adipose tissue and/or an alteration in the hepatic energy metabolism that facilitate intrahepatic fat accumulation. The study conducted by G. Lattuada and collaborators focuses on this clinical aspect. They evaluated the whole-body energy metabolism and hepatic high-energy phosphates in nondiabetic individuals with fatty liver with respect to control individuals matched for anthropometric features. The study analysed the intrahepatic fat content by ^1^H-Magnetic Resonance Spectroscopy, the relative content of hepatic high-energy phosphates (phosphomonoesters, phosphodiesters, inorganic phosphorus, and ATP) by ^31^P-Magnetic Resonance Spectroscopy, and the whole-body resting energy expenditure and substrate oxidation by indirect calorimetry. The authors demonstrated that fasting whole-body energy metabolism and the relative content of hepatic high-energy phosphates in nondiabetic patients with fatty liver are not different than in controls when the two groups of patients are matched for anthropometric features.

F. Yang and collaborators investigated the role of necroptosis during ischemia and reperfusion injury (IRI) in fatty liver. They demonstrated that this cellular process is activated during IRI and that targeting necroptosis can have effects on IRI and ROS production with potential clinical implications.

Experimental data support a role for oxidative stress in the progression of NAFLD toward NASH. These works are well reviewed by M. Masarone and colleagues, who presented the main evidence on the strict pathophysiologic linkage between oxidative stress and NAFLD. Oxidative stress may also affect the synthesis and distribution of gangliosides as reported by V. Šmíd and collaborators. As gangliosides are involved in cell recognition, signalling, and membrane stabilization, the alteration in their expression is often at the basis of many pathological and physiological conditions including cell death, proliferation, and differentiation. Using *in vitro* and *in vivo* models, the authors evaluated the functional consequences of Heme oxygenase 1 deficiency on ganglioside metabolism providing evidence of a tissue-specific increase in the main gangliosides together with changes in the mRNA expression of key enzymes of ganglioside synthesis.

O. Tirosh reviews key mechanisms responsible for the occurrence of NAFLD in lean subjects with a healthy metabolism. In these subjects, the activation of several redox and oxidant signalling pathways involving cholesterol plays a role in fatty liver disease thus indicating that direct lipotoxic effects, more than metabolic alterations, are crucial for the disease progression. Main mechanisms responsible for the cholesterol-induced NAFLD include impairment of the mitochondrial and lysosomal function due to cholesterol loading of the inner cell membrane and the activation of specific signalling and inflammatory pathways. This result has clinical consequences for the development of personal drug and dietary treatment strategies.

Chronic hepatic injury is often related to fibrosis, thus leading to an excessive increase in extracellular matrix protein accumulation and fibrogenesis. Without proper clinical management, liver damage may progress to cirrhosis and ultimately to liver failure or cancer. M. Brancaccio and collaborators put the spotlight on the effect of a marine compound, isolated from sea urchin eggs, as a potential therapeutic molecule for the treatment of liver fibrosis. In particular, they report the effect of ovothiol A, *π*-methyl-5-thiohistidine, on an *in vivo* murine model of liver fibrosis. Interestingly, ovothiol A showed an antifibrotic effect associated with the decrease of fibrogenic markers involved in liver fibrosis progression, such as the transforming growth factor-*β* (TGF-*β*), the *α*-smooth muscle actin (*α*-SMA), and the tissue metalloproteinase inhibitor (TIMP-1). Similarly, in the work of Y. Gao and colleagues, the protective effects of aqueous extracts of *Flos lonicerae Japonicae*, a traditional Chinese medicine, against hydroquinone-induced toxicity were demonstrated using hepatic L02 cells. Aqueous extracts interfere with the production of ROS mediated by hydroquinone, protecting cells from DNA damage and apoptosis.

O. Vázquez-Martínez and collaborators used rat models to evaluate the effect of portacaval anastomosis (PCA) on liver metabolic parameters. Overall, data from their study demonstrated significant liver metabolic and structural adaptations indicating a vascularization process and a reduction of mitochondrial content as consequences of PCA.

Altered cellular metabolism is at the foundation of different liver diseases including HCC. Metabolic pathways that support tumor pathophysiology were summarised by S. De Matteis and collaborators in a comprehensive review article. They discussed how metabolic pathways reprogram liver metabolism to support a specific metabolic demand and how this can be translated into a specific metabolic signature clinically useful for the diagnosis and prognosis of HCC.

Although detailed mechanisms remain to be fully elucidated, several common observations emerge from this special issue pointing to the role that oxidative stress and metabolic pathways may play in liver-associated disorders. Indeed, clinical and biological data clearly show significant differences between normal and pathological conditions. As we are moving toward a systemic classification of human diseases, these metabolic alterations have some practical perspectives in the diagnostic and prognostic clinical field that should be taken into account.

